# Effectiveness of amiodarone versus digitalis for heart rate control in critically ill patients with new-onset atrial fibrillation

**DOI:** 10.1038/s41598-022-06639-0

**Published:** 2022-02-17

**Authors:** Hans-Joerg Gillmann, Philipp Busche, Andreas Leffler, Thomas Stueber

**Affiliations:** Department of Anaesthesiology and Intensive Care Medicine, Hannover Medical School, Carl-Neuberg Strasse 1, 30625 Hannover, Germany

**Keywords:** Medical research, Therapeutics, Adverse effects

## Abstract

New-onset of atrial fibrillation (NOAF) in critically ill patients is the most common acute cardiac dysrhythmia, but evidence-based data regarding treatment strategies are scarce. In this retrospective monocentric study, we compared effectiveness of amiodarone versus digitalis for heart rate control in critically ill patients with new-onset of atrial fibrillation. We identified a total of 209 patients for the main analysis. Amiodarone as compared to digitalis was associated with a clinically relevant faster time to heart rate control < 110 bpm (2 h (IQR: 1 h to 6 h) versus 4 h (2 h to 12 h); p = 0.003) and longer durations of sinus rhythm during the first 24 h of treatment (6 h (IQR: 6 h to 22 h) versus 0 h (IQR: 0 h to 16 h); p < 0.001). However, more bradycardic episodes occurred in association with amiodarone than with digitalis (7.7% versus 3.4%; p = 0.019). Use of amiodarone was associated with an increase of noradrenalin infusion rate compared to digitalis (23.9% versus 12.0%; p = 0.019). Within the tertile of patients with the highest CRP measurements, amiodarone treated patients presented with a higher decrease in heart rate than digoxin treated patients. Clinical trials comparing different NOAF treatment strategies are much needed and should report on concomitant sympathetic activity and inflammatory status.

## Introduction

New-onset of atrial fibrillation (NOAF) is the most common acute cardiac dysrhythmia in critically ill patients. High sympathetic tone due to surgical procedures or critical illness contributes to the high incidence of up to 15% in critically ill patients^1,2^. NOAF may lead to adverse haemodynamic effects, systemic embolism and stroke. In principle two treatment strategies, namely rate or rhythm-control, exist. Existing guidelines for treatment of atrial fibrillation aim at the treatment of atrial fibrillation in the non-critically ill patient^3^. Evidence for the treatment of NOAF in the critically ill patient is scarce. Recent evidence shows comparable outcomes for rhythm and rate control strategies in postoperative NOAF after heart surgery^4^. There are various drugs such as beta blockers, calcium antagonists, digitalis preparations and amiodarone that can be used in intensive care medicine for rate control. Especially in critically ill patients, safety of betablockers and calcium antagonists is discussed controversial due to the risk of hypotensive side effects^5^. Therefore, amiodarone and digitalis are often used in the critically ill patient. In contrast, the effectiveness of digitalis preparations in critically ill patients with high sympathetic tone is unclear due to their vagotonus depended mechanism^6^. There are currently no major comparative studies on the effectiveness of the rate control of digitalis preparations and amiodarone in critically ill patients.

A recent scoping review of treatment strategies for NOAF did not demonstrate any data comparing the effectiveness of digitalis and amiodarone for rate control in NOAF^7^. In a recent multicenter observational study comparing the effect of several medications for rate control in NOAF, fewer than 50 patients treated with digitalis were included^8^. Very recent retrospective data suggest that amiodarone might be preferable for undifferentiated ICU patients with NOAF, but this conclusion also was limited to a small patient sample size^9^.

We therefore conducted this retrospective study to assess the effectiveness of digitalis compared with amiodarone in NOAF in the intensive care unit for heart rate control.

## Methods

### Study design and population

Ethical approval for this retrospective study (Ethical Committee N° 9219_BO_K_2020) was provided by Hannover Medical School Ethics Committee, Hannover, Germany (Chairperson Prof. S. Engeli) on 21st of July 2020, the requirement for informed consent was waived. Research was performed in accordance with relevant guidelines and regulations. This retrospective study included patients admitted to the anaesthesia intensive care unit (ICU, Department of Anaesthesia and Intensive Care Medicine, Hannover Medical School) from January 2015 until July 2020^10^. The anaesthesia intensive care unit is one of six independent surgical intensive care units at Hannover Medical School, a tertiary referral hospital, and provides care for a mixed patient collective (approximately 80% post-surgery, 10% post-interventional and 10% medical cases). There was no dedicated standard operating procedure for management of new-onset of atrial fibrillation, the intensive care physician in charge was responsible for diagnosis, management and choice of medication in these patients.

### Inclusion and exclusion criteria

This study was designed to compare the effectiveness of amiodarone versus digitalis in critically ill patients with NOAF. We therefore included patients (1) with NOAF as diagnosed by the treating physician, (2) no prior treatment with amiodarone or digitalis, (3) with an ICU treatment duration of at least 24 h and (4) aged 18 years or older. Patients were excluded if (1) admitted repeatedly (only the most recent stay in the study interval was evaluated), (2) long-standing persistent atrial fibrillation was documented or clinically suggested, (3) ICU stay was shorter than 6 h after atrial fibrillation treatment initiation, (4) patients underwent left ventricular assist device or extracorporal membrane oxygenation therapy, (5) cardioversion was executed after digitalis or amiodarone therapy (exclusion from the main analysis because of interference with the effect of the medication; data are reported in the supplement), (6) patients received both amiodarone and digitalis within 72 h, or (7) if the treating physician chose a treatment strategy without amiodarone and digitalis.

### Data collection

Treatment on the study ICU was documented using the patient data management system (PDMS) m.life (medisite GmbH, Hannover, Germany). Patient data (anthropometric and baseline data, medication, medical history, vital parameters, ICU data) were extracted manually into a Microsoft Excel based (Microsoft Corporation, Redmond, WA) study work sheet with this data being stored on a secured intrahospital server. After completion and cross-checking of these work sheets, identifying patient data were deleted and afterwards exported to SPSS (SPSS, Chicago, IL) for anonymized data analyses. Patient heart rates and other vital parameters are documented within the PDMS every minute.

### Study endpoints and main outcome measures

As we aimed to compare the effectiveness of amiodarone versus digitalis for heart rate control, the main outcome measure for this study was defined as the time from amiodarone or digitalis start to reaching a heart rate of < 110 beats per minute (bpm) for at least 1 h. According to prospective trial data, heart rate control at < 110 bpm is equivalent to rhythm control in terms of clinical outcome^11^. Representative readings of heart rates were documented one hour prior to medication with amiodarone or digitalis as well as three, six, twelve, 24, 48 and 72 h afterwards (as long as the patient was still treated in the ICU). As continuous electrocardiogram raw data were not available for this retrospective data, we inferred occurrence of conversion from atrial fibrillation into sinus rhythm from an abrupt characteristic change in heart rate.

Secondary outcomes measures were (1) heart rate delta relative to medication start and (2) hours in sinus rhythm within the first 24 h after medication.; (3) hours until sinus rhythm conversion (up to 72 h), (4) vital parameters at sinus rhythm conversion, (5) changes in hemodynamic stability associated with AF treatment as measured by change in vasopressor dosing and (6) occurrence of bradycardic episodes (heart rate < 50 bpm for > 10 min). Additionally, we planned subgroup analyses of these endpoints for septic patients, stratified both by clinical diagnosis and by C-reactive protein (CRP) levels. Exploratory analyses for patients undergoing electrical cardioversion are reported in the Supplementary Appendix.

### Statistical analysis

We did not identify comparative studies investigating amiodarone versus digitalis for new-onset atrial fibrillation in critically ill patients. Herasevich et al. reported that 24 h after digitalis medication, the majority of patients reached sufficient heart rate control^12^. Because of the explorative design of this study, we aimed to include the largest sample size possible from the study ICU database. We were only able to extend the maximum recruitment interval back to January 2015 because of a change in the PDMS variable architecture at this time point.

Data are presented as medians with their respective interquartile range (IQR) or as means and 95% confidence interval as appropriate. Data were tested non-parametrically, because heart rate data are distributed non-normally. Multiple groups were compared non-parametrically with the Kruskal–Wallis test, two-group comparisons with the Mann–Whitney U test. Paired data were compared with the Friedman test. Global level of significance was set at a p-value of less than 0.05. The Bonferroni-Holm correction was used for subgroup comparisons^13^. Patients with missing heart rate data were excluded from analysis of only the respective time point. Patients with a heart rate of < 110 bpm without treatment were excluded from primary endpoint analysis. Propensity score matching was used to correct for differences in heart rate at NOAF treatment initiation. For the subgroup analysis stratified by CRP, we calculated tertiles of CRP measurements. Data were analysed with SPSS (SPSS, Chicago, Illinois, USA) and MedCalc (MedCalc Software, Ostende, Belgium).

## Results

### Patient characteristics

**Figure 1:**
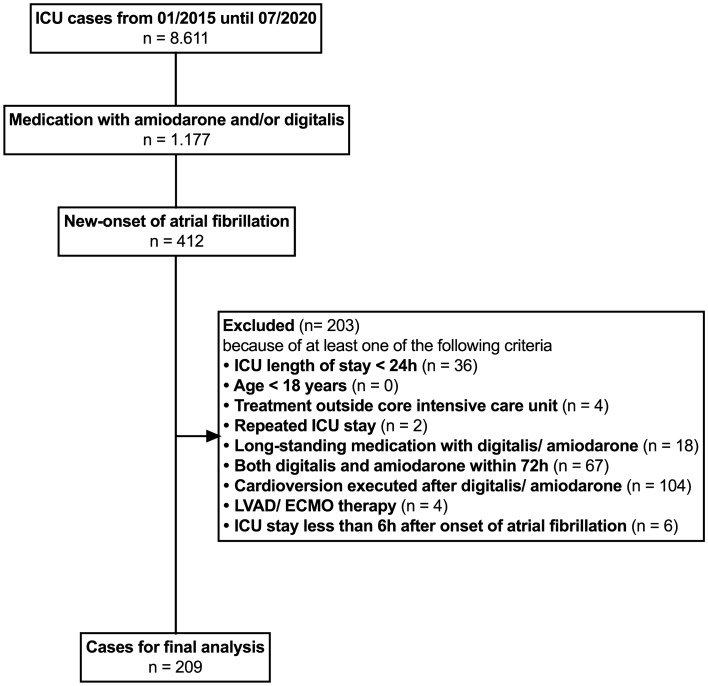
Study flow chart. ICU, intensive care unit; ECMO, extracorporeal membrane oxygenation; LVAD, left-ventricular assist device. Study flowchart showing the number of patients and respective exclusion criteria. Patients receiving both amiodarone and digitalis were excluded from primary endpoint analysis for methodical reasons. Patients in need of a cardioversion after medication were also excluded, because we intended to examine solely the effectiveness of amiodarone or digitalis medication for heart rate control. Patients with an ICU stay of less than 6 h after onset of atrial fibrillation were excluded, because we deemed this time interval as an insufficient follow-up for the study.

All 8611 admissions to the ICU between January 2015 and July 2020 were screened for this study (Fig. 1). 1177 (13.5%) patients were identified with a documented medication of intravenous amiodarone or digitalis and underwent individual patient chart data review. 412 (4.8%) patients were identified with new-onset of atrial fibrillation. Of these, 103 patients were treated with electrical cardioversion following amiodarone or digitalis (amiodarone: 38%, digitalis 11%; p < 0.001; see Supplement). Because of interference with effectiveness estimation of amiodarone or digitalis, these patients were excluded from the main analysis. Data for patients undergoing electrical cardioversion can be found in the Supplementary Appendix (Supplementary Tables S1 to S3). Consecutively, 209 patients remained for the final analysis dataset (Fig. 1). Main results are reported for 92 patients that were treated with amiodarone only and for 117 patients that received digitalis medication only. Baseline characteristics of the included patients are shown in Table 1. Patients receiving amiodarone for new-onset of atrial fibrillation presented with a higher illness severity as compared to patients receiving digitalis (SAPS-II at admission 48 versus 42, p = 0.012). Median doses within the first 24 h after treatment start were 0.6 mg [IQR: 0.4 to 0.8 mg] digitoxin, 0.8 mg [IQR: 0.5 to 1.0 mg] digoxin or 300 mg [IQR: 300 to 1000 mg] amiodarone. Within 72 h after treatment start, median doses were 0.9 mg [IQR 0.7 to 1.1 mg] digitoxin, 1.1 mg [IQR: 0.8 to 1.6 mg] digoxin or 500 mg (300 to 1450 mg) amiodarone.

**Table 1:** Baseline characteristics of the patients.

Quantitative parameters	Total (n = 209)	Amiodarone (n = 92)	Digitalis (n = 117)	p-value
	Median [IQR]	Median [IQR]	Median [IQR]	
Age (y)	74 [65 to 80]	72 [63 to 80]	76 [67 to 80]	0.050
Weight (kg)	76 [68 to 90]	75 [68 to 90]	77 [68 to 90]	0.816
Height (cm)	170 [165 to 180]	170 [164 to 180]	170 [165 to 180]	0.966
SAPS-II at admission	45 [36 to 54]	48 [38 to 56]	42 [34 to 51]	**0.012**
ICU day with onset of AF	0 [0 to 3]	1 [0 to 7]	0 [0 to 2]	** < 0.001**

Qualitative parameters	Total (n = 209)	Amiodarone (n = 92)	Digitalis (n = 117)	p-value
	% (n)	% (n)	% (n)	
Gender male	59 (123)	58 (39)	60 (70)	0.747
Admission postoperative	59 (124)	59 (54)	60 (70)	0.869
Admission b/o Stroke	22 (46)	16 (15)	27 (31)	0.078
Admission b/o ICB	6 (13)	4 (4)	8 (9)	0.321
CAD	27 (57)	27 (25)	27 (32)	0.836
CHF	27 (56)	24 (22)	29 (34)	0.970
Stroke	19 (39)	22 (20)	16 (19)	0.423
CKD	23 (47)	25 (23)	21 (24)	0.446
COPD	13 (27)	10 (9)	15 (18)	0.560
Diabetes	21 (43)	25 (23)	17 (20)	0.164
Sepsis	26 (5)	33 (30)	21 (25)	0.068

AF, atrial fibrillation; CAD, coronary heart disease; CHF, chronic heart failure; CKD, chronic kidney disease; COPD, chronic obstructive pulmonary disease; ICB, intracranial bleeding; ICU, intensive care unit; IQR, interquartile range; SAPS, simplified acute physiology score.

p-value: Mann–Whitney U test for the two subgroups. New onset of atrial fibrillation in patients receiving amiodarone occurred (median) on ICU day 1 as compared to patients receiving digitalis (day 0), but this was judged as clinically nonrelevant. Patients receiving amiodarone presented with a higher SAPS-II score at ICU admission, pointing at a higher illness severity in patients receiving amiodarone.

Significant values are in bold.

### Heart rate control earlier with amiodarone

**Figure 2:**
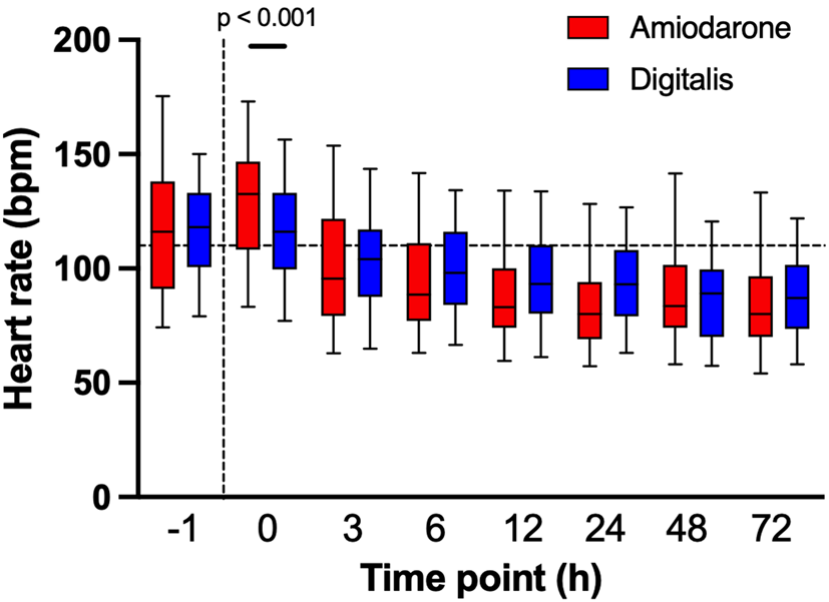
Heart rate decreases over time. Bpm, beats per minute; h, hour. Boxplots (whiskers: 5th and 95th percentile) show representative heart rates for patients treated with amiodarone (red) or digitalis (blue) during the study interval. The horizontal line marks 110 bpm, the vertical line represents medication start. Heart rates both in patients treated with either amiodarone or digitalis decreased over time (Friedman test: p < 0.001 for both groups). Median heart rates both in amiodarone and digitalis treated patients remained consistently lower than at study start (Bonferroni-Holm corrected p < 0.01 for each time point versus 0 h). Patients receiving amiodarone presented with a higher heart rate at 0 h (133 bpm [IQR: 109 bpm to 147 bpm] versus 116 bpm [IQR: 100 bpm to 133 bpm]; p < 0.001).

Patients receiving amiodarone or digitalis presented with a consistent decrease in heart rates during the 72 h study interval (Friedman test for both groups p < 0.001; Fig. 2). 132 patients presented with a heart rate of > 110 bpm at medication start. Within this group, time to heart rate control < 110 bpm was reached earlier in patients receiving amiodarone as compared to digitalis (2 h [IQR: 1 h to 6 h] versus 4 h (2 h to 12 h); p = 0.003). Two patients treated with amiodarone versus one patient treated with digitalis never reached heart rate control < 110 bpm within 72 h after treatment initiation (p = 0.427).

Thirteen (14%) patients treated with amiodarone versus 66 (56%) patients treated with digitalis never reached sinus rhythm within 72 h after treatment initiation (p < 0.001).

At medication start, patients receiving amiodarone presented with a higher heart rate than patients receiving digitalis (133 bpm [IQR: 109 bpm to 147 bpm] versus 116 bpm [IQR: 100 bpm to 133 bpm]; p < 0.001). However, in a propensity score analysis (propensity score caliper value 0.05, heart rate at NOAF treatment initiation as a factor), the more effective heart rate reduction in association with amiodarone remained statistically significant (Table 2).

**Table 2:** Propensity score matched comparison of heart rates.

Time point	Amiodarone (n = 84)	Digitalis (n = 84)	p-value
	bpm median [IQR]	bpm median [IQR]	
1 h prior	116 [91 to 137]	119 [102 to 135]	0.510
0 h	129 [106 to 143]	125 [103 to 138]	** < 0.001**
3 h	99 [81 to 122]	105 [92 to 121]	1.000
6 h	90 [77 to 111]	99 [84 to 117]	0.510
12 h	83 [74 to 100]	94 [81 to 112]	**0.013**
24 h	80 [69 to 94]	95 [79 to 112]	**0.004**
48 h	84 [73 to 103]	89 [72 to 100]	0.883
72 h	80 [70 to 94]	87 [74 to 102]	0.719

bpm, beats per minute; IQR, interquartile range; h, hours.

168 patients could be matched using propensity score matching (caliper value 0.05; heart rate at 0 h as a factor). p-value: Wilcoxon test for the two matched subgroups. A stricter propensity score matching caliper value did not change the pattern of significant differences between the amiodarone and digitalis group. We detected clinically relevant lower heart rates in patients treated with amiodarone as compared to digitalis starting 12 h and 24 h after treatment. This difference diminished afterwards.

Significant values are in bold.

### More effective heart rate reduction with amiodarone

**Figure 3:**
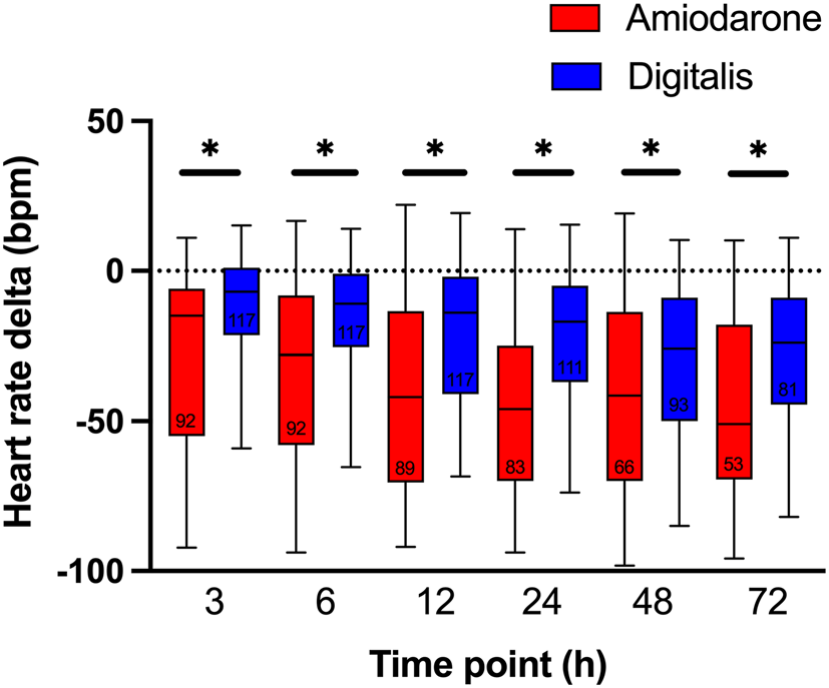
Heart rate delta over time. Bpm, beats per minute; h, hour. Boxplots (whiskers: 5th and 95th percentile) show representative heart rates for patients treated with amiodarone (red) or digitalis (blue) during the study interval. * indicates Mann–Whitney U p-values < 0.05 (Bonferroni-Holm corrected for comparison of 6 subgroups). Numbers within the boxes represent the included patients for this time point. Patients receiving amiodarone presented with a greater relative decrease in heart rate at any measured time point within the study interval.

Amiodarone as well as digitalis treated patients presented with a progressive reduction in heart rate during the 72 h study interval (Friedman test for both groups p < 0.001, Fig. 3). Maximum relative reduction in heart rate was detected 72 h after treatment start in amiodarone treated patients (− 51 bpm; IQR: − 70 bpm to − 18 bpm) and 48 h after treatment start in digitalis treated patients (− 26 bpm; IQR: − 50 bpm to − 9 bpm). 3 h after treatment initiation, amiodarone treated patients reached a median reduction of − 15 bpm versus − 7 bpm in digitalis treated patients (p < 0.001). Subgroup comparisons in amiodarone treated patients showed a continuous additional reduction in median heart rates until 12 h after treatment start (− 42 bpm; p = 0.003). In comparison, heart rates in digitalis treated patients did not significantly decrease further 6 h after treatment (6 h: − 11 bpm, p = 0.004; 12 h: − 14 bpm, p = 0.143; Fig. 3). Relative reduction in heart rates was almost twice as high in amiodarone treated patients as in digitalis treated patients (p < 0.05 for heart rate delta, comparing amiodarone versus digitalis; Fig. 3).

### Secondary outcome measures

Patients treated with amiodarone had a significantly longer duration of hours in sinus rhythm than digitalis treated patients within 24 h of treatment initiation (6 h [IQR: 6 h to 22 h] versus 0 h [IQR: 0 h to 16 h]; p < 0.001). Regarding the time point of first conversion from atrial fibrillation into sinus rhythm, we did not find a difference between amiodarone and digitalis treated patients (3 h versus 2 h; p = 0.755). Systolic blood pressures and mean arterial blood pressures did not differ at any of the recorded time points. Similarly, at conversion into sinus rhythm, systolic blood pressures (amiodarone: 123 mmHg [IQR: 109 mmHg to 135 mmHg]; digitalis: 120 mmHg (107 mmHg to 133 mmHg); p = 0.645) and mean arterial blood pressure (amiodarone: 80 mmHg [IQR: 69 mmHg to 91 mmHg]; digitalis: 78 mmHg [IQR: 73 mmHg to 86 mmHg]; p = 0.605) did not significantly differ. 32% of all patients presented with decreasing norepinephrine infusion rates during the study interval, whereas amiodarone versus digitalis treatment was associated with a higher need of increased norepinephrine infusion rates (23.9% versus 12.0%; p = 0.019). Bradycardic episodes (heart rate < 50 bpm for > 10 min) within 72 h after treatment initiation occurred more frequently in amiodarone treated patients than in digitalis treated patients (7.7% versus 3.4%; p = 0.019).

Considering additional comedications for heart rate control, 108 patients received additional beta receptor blockers, but without a significant distributional difference between amiodarone and digitalis treated patients (p = 0.325). Similarly, 25 patients received additional calcium antagonists, but also not significantly different between the both groups (p = 0.086).

Electrical cardioversion after medication led to conversion into sinus rhythm in 24 (23%) patients. With regard to cardioversion success, amiodarone was associated with an increased cardioversion success (amiodarone 32% versus digitalis 13%; p = 0.019; Supplementary Table S3). Incidence of post-shock bradycardia was not statistically different in patients receiving amiodarone, digitalis or both medications within 24 h (p = 0.350) and 72 h after treatment initiation (p = 0.554), but this analysis was likely underpowered (Supplementary Table S3 and Fig. S2).

### Higher effectiveness in septic patients and hyperinflammation with amiodarone

**Figure 4:**
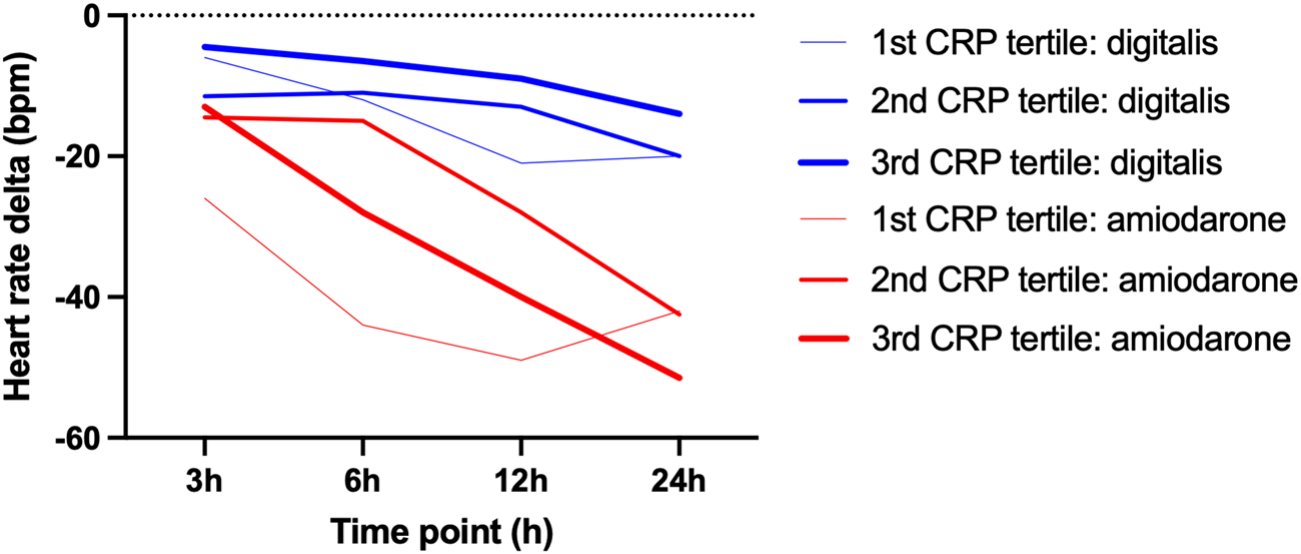
Association of heart rate control with inflammation. Bpm, beats per minute; CRP, C-reactive protein; h, hour. Patients are grouped by amiodarone (red) versus digitalis (blue) and CRP tertile (1st: < 143 mg/L; 2nd: 143 to 263 mg/L; 3rd: > 263 mg/L). Comparison of the tertile lines shows that increasing CRP values were associated with greater heart rate decrease in association with amiodarone treatment than with digitalis treatment (univariate ANOVA: p < 0.001 both for the factors CRP tertile and medication group).

Fifty-five (26.3%) of the included patients were diagnosed with sepsis (27 (49%) pulmonary; 14 (25%) abdominal; 16 (29%) urogenital; 20 (36%) bacteremia; more than one focus possible). Stratifying patients by CRP tertile, amiodarone treated patients presented with a higher decrease in heart rate than digoxin treated patients at any registered time point and especially in those patients with the highest CRP measurements (Fig. 4).

## Discussion

In our retrospective study, amiodarone in comparison to digitalis was associated with a clinically significant faster decrease in heart rate, when treating NOAF in critically ill patients. During the 72 h study interval starting with initiation of treatment, amiodarone was associated with an absolute heart rate reduction of 10 bpm more than digitalis and a relative reduction in heart rate twice as effective as digitalis. Patients treated with amiodarone had significantly longer time intervals with sinus rhythm during the first 24 h of treatment, but bradycardic episodes occurred more frequent with amiodarone than with digitalis. Use of digitalis was associated with more frequent relapses of atrial fibrillation than amiodarone as quantified by the cumulative time in sinus rhythm during the first 24 h of treatment initiation. Amiodarone more than digitalis seemed to improve likelihood of successful electrical cardioversion. Considering CRP as a marker of inflammatory stress, amiodarone was associated with faster heart rate control than digitalis in patients with the highest CRP values.

Drikite et al. reported a clinically relevant heterogeneity even in definition of NOAF, presenting differences in the defining heart rate and duration of atrial fibrillation^7^. Because we identified NOAF from patients’ charts and characteristic change patterns in heart rates as documented in the PDMS, we may have missed patients with very short or nearly regular ventricular rate episodes of atrial fibrillation. However, our detected frequency of NOAF around 5% was comparable to studies published by Seguin et al. in trauma patients and McKenzie Brown et al. in non-cardiac post-operative patients^14^. Seguin et al. also reported a frequency of cardioversions that was comparable to our collective, especially considering the proportion of patients receiving electrical cardioversion. Because we intended to examine the pharmacologic effects of amiodarone versus digitalis in heart rate and rhythm control for NOAF, we excluded patients electrically cardioverted after medication from primary data analysis to reduce bias.

In our study, both amiodarone and digoxin resulted in clinically relevant slower heart rates. Amiodarone may be more effective with regard to conversion into sinus rhythm and heart rate slowing. Effectiveness of heart rate control in digitalis treated patients and digitalis dosing were comparable to study data by Herasevich et al., who reported a decrease in heart rate of 23 bpm followed by a slight increase in systolic blood pressures^12^. McKenzie Brown et al. reported amiodarone as the most efficient pharmacologic intervention to reach heart rate and rhythm control, which also is compatible with our findings^15^.

Dosing of amiodarone and digitalis preparations in our cohort may have influenced the effectiveness of heart rate control. Usual dosing for digoxin is a loading dose of around 1 mg in the first 24 h and for amiodarone approximately 1 g in the initial 24 h. The median dosing of amiodarone in our cohort (300 mg compared to 1 g within 24 h) was relatively lower than the digitalis dosing in our cohort (0.8 mg compared to 1 mg within 24 h) when compared to usual dosing. This may have caused more likely caused an underestimation of amiodarone effect rather than an overestimation.

Comparison of effectiveness in this study may be also influenced by bias due to the retrospective design of the study. Physicians might have chosen to use amiodarone preferably to digoxin in sicker patients or based on other patient characteristics. However, our finding of an increased effectiveness for amiodarone versus digitalis in ICU patients with NOAF is in line with the recently published data from Bedford et al. and emphasizes the need for consideration of physiological status in NOAF treatment^9^. Although we assessed effectiveness of treatment with respect to heart rate, one must bear in mind, that this is only one variable with respect to treatment of atrial fibrillation. The purpose of medications in acute onset atrial fibrillation is to improve cardiac output, which is not only dependent on heart rate. However, optimal heart rate in atrial fibrillation may increase blood pressure, but a large reduction in heart rate due to the antiarrhythmic treatment may induce hypotension. The optimal heart rate to maximize cardiac output in a critically ill patients is currently unknown but may well be faster than 100 bpm**.**

We did not find an increase in blood pressure after rhythm or rate control but a decrease in vasopressor dosing for 32% of the included patients, while Herasevich et al. reported unchanged vasopressor needs. This might be explained by a difference in the included patient collectives between the two studies. The use of amiodarone was associated with an 12% absolute increase in the need for a higher vasopressor and dose compared to digitalis in our study. If this effect was due to a slower heart rate, an intrinsic hypotensive effect of amiodarone or due to bias by indication cannot be concluded from this retrospective analysis. The use of amiodaron was also associated with an increase in the incidence of bradycardia.

Due to the retrospective design of our study, we detected relevant baseline differences associated with amiodarone versus digitalis treated patients. First, patients treated with amiodarone had higher simplified acute physiology scores (SAPS-II) at admission than digitalis treated patients. Second, patients treated with amiodarone also presented with higher heart rates at atrial fibrillation treatment initiation. We cannot exclude bias in treatment selection based on initial heart rate, renal function, severity of hemodynamic instability, presence or absence of heart failure as well as expectation of effectiveness or adverse effects. Our findings of a more effective heart rate control associated with amiodarone however remained clinically relevant even after propensity score matching for the initial heart rate. Acknowledging these limitations, our study confirms recent retrospective studies and to our knowledge is the largest database for direct comparison of amiodarone versus digitalis treatment in ICU patients with NOAF. Given that NOAF is a frequent complication associated with worse clinical and patient-centered outcome, clinical trials comparing the effectiveness of treatments such as amiodarone and digitalis will add essential information towards evidence-based decision making in atrial fibrillation treatment of the critically ill. Future randomised trials of NOAF treatment in ICU patients may document sympathetic activity and inflammatory status of the included patients for identification of important subgroups.

In conclusion, treatment with either amiodarone or digitalis was associated with a relevant decrease in ventricular rate in critically ill patients with new onset of atrial fibrillation. Our data indicated, that treatment with amiodaron had a faster blocking effect on the AV node and the effectiveness of heart rate control was higher compared to digitalis. However, this also was associated with a higher incidence of hypotension and bradycardia. There is a need for randomized controlled trials comparing the risk and benefits of amiodarone and digitalis in critically ill patients with atrial fibrillation.

## Supplementary Information

Supplementary Information.

## References

[1] Klouwenberg PMCK, Frencken JF, Kuipers S, Ong DSY, Peelen LM, Vught LAV (2017). Incidence, predictors, and outcomes of new-onset atrial fibrillation in critically ill patients with sepsis. A cohort study. Am. J. Respir. Crit. Care Med..

[2] Yoshida T, Fujii T, Uchino S, Takinami M (2015). Epidemiology, prevention, and treatment of new-onset atrial fibrillation in critically ill: A systematic review. J. Intensive Care..

[3] Hindricks G, Potpara T, Dagres N, Arbelo E, Bax JJ, Blomström-Lundqvist C (2020). 2020 ESC Guidelines for the diagnosis and management of atrial fibrillation developed in collaboration with the European Association for Cardio-Thoracic Surgery (EACTS): The Task Force for the diagnosis and management of atrial fibrillation of the European Society of Cardiology (ESC) Developed with the special contribution of the European Heart Rhythm Association (EHRA) of the ESC. Eur. Heart J..

[4] Gillinov AM, Bagiella E, Moskowitz AJ, Raiten JM, Groh MA, Bowdish ME (2016). Rate control versus rhythm control for atrial fibrillation after cardiac surgery. N. Engl. J. Med..

[5] Labbé V, Bagate F, Cohen A, Voiriot G, Fartoukh M, Mekontso-Dessap A (2021). A survey on the management of new onset atrial fibrillation in critically ill patients with septic shock. J. Crit. Care..

[6] Watanabe AM (1985). Digitalis and the autonomic nervous system. J. Am. Coll. Cardiol..

[7] Drikite L, Bedford JP, O’Bryan L, Petrinic T, Rajappan K, Doidge J (2021). Treatment strategies for new onset atrial fibrillation in patients treated on an intensive care unit: A systematic scoping review. Crit. Care.

[8] Bosch NA, Rucci JM, Massaro JM, Winter MR, Quinn EK, Chon KH (2021). Comparative effectiveness of heart rate control medications for the treatment of sepsis-associated atrial fibrillation. Chest.

[9] Bedford JP, Johnson A, Redfern O, Gerry S, Doidge J, Harrison D (2022). Comparative effectiveness of common treatments for new-onset atrial fibrillation within the ICU: Accounting for physiological status. J. Crit. Care..

[10] Stueber T, Buessecker L, Leffler A, Gillmann HJ (2017). The use of dipyrone in the ICU is associated with acute kidney injury: A retrospective cohort analysis. Eur. J. Anaesthesiol..

[11] Van Gelder IC, Groenveld HF, Crijns HJ, Tuininga YS, Tijssen JG, Alings AM (2010). Lenient versus strict rate control in patients with atrial fibrillation. N. Engl. J. Med..

[12] Herasevich S, Bennett CE, Schwegman AR, Subat YW, Gajic O, Jayaprakash N (2019). Hemodynamic profiles following digoxin use in patients with sepsis in the ICU. J. Crit. Care..

[13] Holm S (1979). A simple sequentially rejective multiple test procedure. Scand. J. Stat..

[14] Seguin P, Laviolle B, Maurice A, Leclercq C, Mallédant Y (2006). Atrial fibrillation in trauma patients requiring intensive care. Intensive Care Med..

[15] Brown M, Nassoiy S, Chaney W, Plackett TP, Blackwell RH, Luchette F (2018). Impact and treatment success of new-onset atrial fibrillation with rapid ventricular rate development in the surgical intensive care unit. J. Surg. Res..

