# Supplementary material for: Effectiveness of amiodarone versus digitalis for heart rate control in critically ill patients with new-onset atrial fibrillation
Source: Sci Rep. 2022 Feb 17;12:2712. doi: 10.1038/s41598-022-06639-0 (PMC8854600; doi:10.1038/s41598-022-06639-0)
Supplement: Supplementary file 1 — Supplementary Information. [file 41598_2022_6639_MOESM1_ESM.pdf]

SUPPLEMENTARY INFORMATION

Effectiveness of amiodarone versus digitalis for heart rate control in critically ill patients with new-onset atrial fibrillation

Hans-Joerg Gillmann, MD[a]; Philipp Busche[a], Andreas Leffler, MD[a]; Thomas Stueber, MD[a]

[a]Department of Anaesthesiology and Intensive Care Medicine, Hannover Medical School, Hannover, Germany

Supplemental Figure 1. Study flow chart (all patients including those with electrical cardioversion after medication)

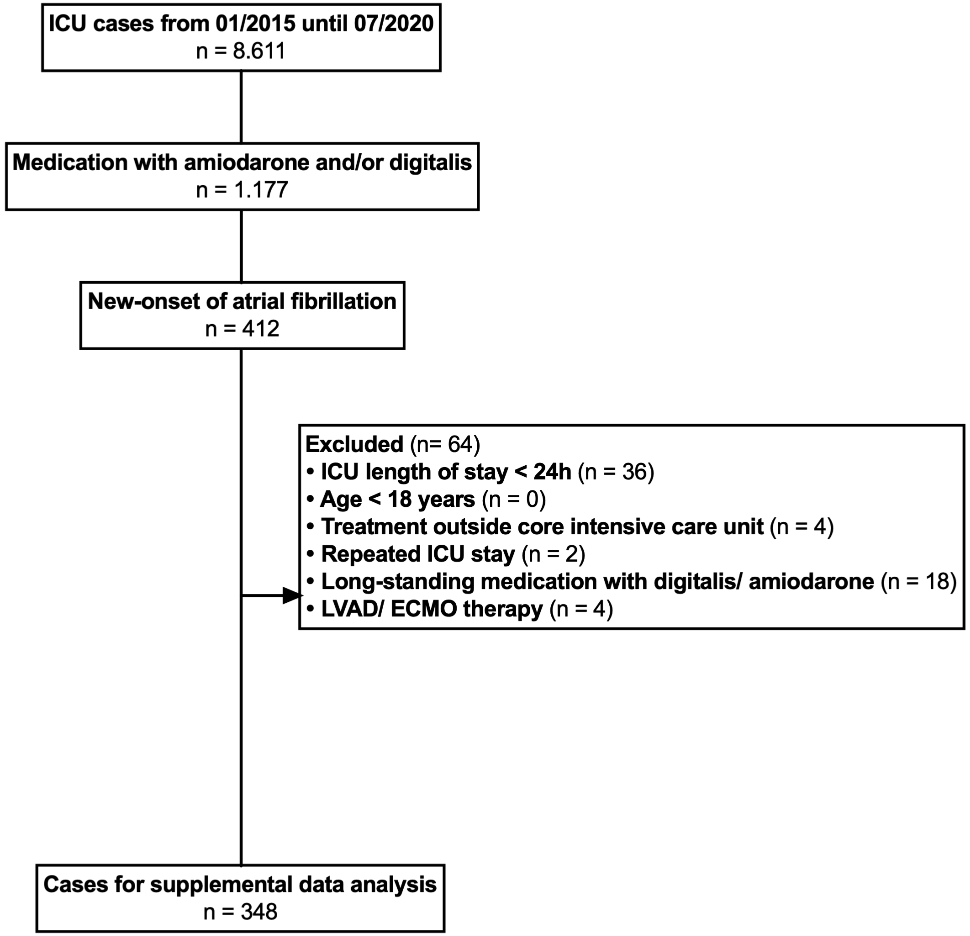

ICU, intensive care unit; ECMO, extracorporeal membrane oxygenation; LVAD, left-ventricular assist device. Study flowchart showing the number of patients and respective exclusion criteria. For supplemental exploratory data analyses, additional patients were included (patients (1) receiving electrical cardioversion, (2) receiving both amiodarone and digitalis). This resulted in 348 patients included into supplemental analyses.

Supplemental Figure 2. Heart rate decreases over time (incl. patients receiving electrical cardioversion and both medications)

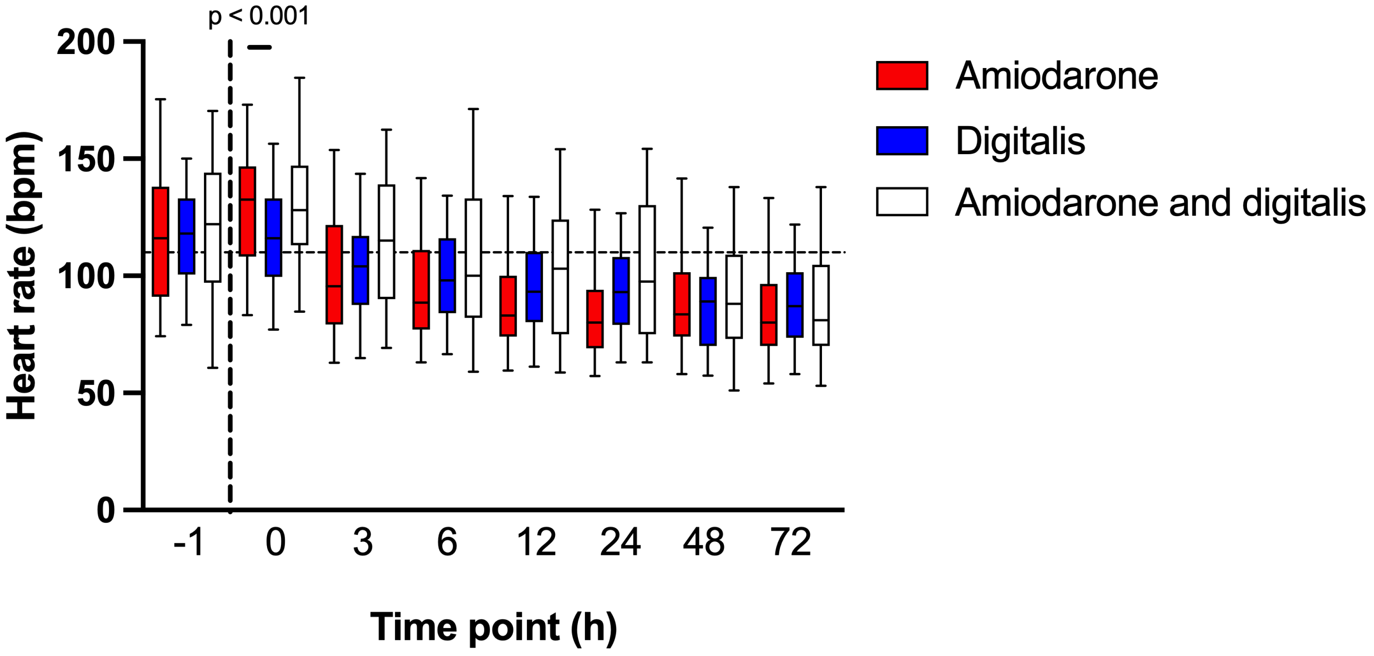

Bpm, beats per minute; h, hour. Boxplots (whiskers: 5th and 95th percentile) show representative heart rates for patients treated with amiodarone (red), digitalis (blue) or both medications (white) during the study interval. The horizontal line marks 110 bpm, the vertical line represents medication start. Heart rates both in patients treated with either amiodarone, digitalis or both medications decreased over time (Friedman test: $p < 0.001$ for the three groups). Median heart rates in the three presented groups were consistently lower than at study start, except at the time point three hours after digitalis (uncorrected p=0.0248; not significant using Bonferroni-Holm correction $p < 0.01$ for each time point versus 0 hours) or both medications (uncorrected p=0.1660). Patients receiving amiodarone presented with a higher heart rate at 0h than digitalis treated patients (133 bpm [IQR: 109 bpm to 147 bpm] versus 116 bpm [IQR: 100 bpm to 133 bpm]; adjusted $p < 0.035$). Patients treated both with amiodarone and digitalis presented with a delayed decrease in heart rates as compared to patients treated with either amiodarone or digitalis alone. This finding is prone to uncontrolled confounding factors (i.e. clinical reasoning for treating patients both with amiodarone and digitalis).

Supplemental Table 1. Baseline Characteristics of the Patients

Quantitative Parameters	Total (n=348) Median [IQR]	Amiodarone (n=149) Median [IQR]	Digitalis (n=132) Median [IQR]	Amiodarone and Digitalis (n=67) Median [IQR]	Electrical cardioversion after medication (n=103) Median [IQR]
Age (y)	73 [64 to 80]	71 [62 to 79]	75 [67 to 80]	74 [66 to 80]	70 [61 to 78]
Weight (kg)	77 [68 to 90]	80 [70 to 90]	75 [67 to 89]	78 [63 to 85]	78 [70 to 90]
Height (cm)	172 [165 to 180]	174 [165 to 180]	172 [165 to 180]	170 [165 to 175]	175 [170 to 180]
SAPS-II at admission	47 [37 to 55]	49 [37 to 57]	42 [35 to 51]	48 [41 to 58]	48 [37 to 57]
ICU day with onset of AF	1 [0 to 3]	1 [0 to 4]	0 [0 to 1]	0 [0 to 2]	1 [0 to 3]

ICU, intensive care unit; IQR, interquartile range; SAPS, simplified acute physiology score. New onset of atrial fibrillation in patients receiving amiodarone occurred (median) on ICU day 1 as compared to patients receiving digitalis (day 0), but this was judged as clinically nonrelevant. Patients receiving digitalis presented with a lower SAPS-II score at ICU admission than the other shown groups, pointing at a lower illness severity in patients receiving digitalis.

Supplemental Table 2. Baseline Characteristics of the Patients					
**Qualitative Parameters**	**Total (n=348)**	**Amiodarone (n=149)**	**Digitalis (n=132)**	**Amiodarone and Digitalis (n=67)**	**Electrical cardioversion after medication (n=103)**
	% (n)	% (n)	% (n)	% (n)	% (n)
**Gender male**	62 (215)	65 (97)	61 (80)	57 (38)	74 (76)
**Admission postoperative**	54 (188)	54 (81)	60 (78)	43 (29)	50 (51)
**Admission b/o Stroke**	20 (70)	12 (18)	26 (34)	27 (18)	11 (11)
**Admission b/o ICB**	6 (22)	5 (7)	8 (10)	8 (5)	6 (6)
**CAD**	25 (88)	26 (39)	28 (37)	18 (12)	26 (26)
**CHF**	25 (86)	24 (35)	29 (38)	19 (13)	23 (24)
**Stroke**	18 (63)	18 (27)	16 (21)	22 (15)	17 (17)
**CKD**	22 (76)	27 (40)	20 (26)	15 (10)	23 (24)
**COPD**	12 (43)	11 (16)	15 (20)	10 (7)	14 (14)
**Diabetes**	23 (79)	29 (43)	17 (22)	21 (14)	26 (27)
**Sepsis**	31 (107)	37 (55)	23 (30)	33 (22)	40 (41)

AF, atrial fibrillation; CAD, coronary heart disease; CHF, chronic heart failure; CKD, chronic kidney disease; COPD, chronic obstructive pulmonary disease; ICB, intracranial bleeding. Patients receiving both amiodarone and digitalis were more often admitted for medical reasons and presented with a higher incidence of prior stroke than the other presented subgroups.

Supplemental Table 3. Endpoints

Quantitative Parameters	Total (n=348)	Amiodarone (n=149)	Digitalis (n=132)	Amiodarone and Digitalis (n=67)	Electrical cardioversion after medication (n=103)
	Median [IQR]	Median [IQR]	Median [IQR]	Median [IQR]	Median [IQR]
Hours within sinus rhythm during the first 24h	13 [0 to 20]	17 [7 to 21]	0 [0 to 17]	8 [0 to 18]	17 [6 to 20]
Hours until heart rate < 110 bpm	2 [0 to 5]	1 [0 to 4]	2 [0 to 6]	3 [1 to 9]	2 [0 to 5]
**Qualitative Parameters**	**Total (n=348)**	**Amiodarone (n=92)**	**Digitalis (n=117)**	**Amiodarone and Digitalis (n=67)**	**Electrical cardioversion after medication (n=103)**
	% (n)	% (n)	% (n)	% (n)	% (n)
**Electrical cardioversion after medication**	30 (103)	38 (57)	11 (15)	46 (31)	-- (--)
**Successful electrical cardioversion after medication**	23 (24)	32 (18)	13 (2)	13 (4)	-- (--)
**Incidence of post-shock bradycardia within 24h after treatment initiation**	5 (16)	4 (6)	5 (6)	6 (4)	6 (6)
**Incidence of post-shock bradycardia within 72h after treatment initiation**	15 (52)	17 (25)	8 (11)	24 (16)	17 (17)
**Never sinus rhythm within 72h**	29 (100)	9 (13)	53 (70)	25 (17)	8 (8)
**Never heart rate <110 bpm within 72h**	2 (6)	1 (1)	2 (2)	5 (3)	0 (0)

Bpm, beats per minute; IQR, interquartile range. Electrical cardioversion after medication led to conversion into sinus rhythm in 24 (23%) patients. With regard to cardioversion success, amiodarone was associated with an increased cardioversion success (amiodarone 32% versus

digitalis 13%; $p=0.019$). Incidence of post-shock bradycardia was not statistically different in patients receiving amiodarone, digitalis or both medications within 24 hours ($p=0.350$) and 72 hours after treatment initiation ($p=0.554$), but this analysis was likely underpowered.
